# Socioeconomic disparities in immunotherapy use among advanced-stage non-small cell lung cancer patients: analysis of the National Cancer Database

**DOI:** 10.1038/s41598-023-35216-2

**Published:** 2023-05-20

**Authors:** Anjali Gupta, Chioma Omeogu, Jessica Y. Islam, Ashwini Joshi, Dongyu Zhang, Dejana Braithwaite, Shama D. Karanth, Tina D. Tailor, Jeffrey M. Clarke, Tomi Akinyemiju

**Affiliations:** 1 Department of Population Health Sciences, Duke University School of Medicine, 215 Morris Street, Durham, NC 27708 USA; 2 Cancer Epidemiology Program, H. Lee Moffitt Cancer Center and Research Institute, Tampa, FL USA; 3 Johnson and Johnson, Medical Device Epidemiology, New Brunswick, NJ USA; 4 Department of Epidemiology, University of Florida, Gainesville, FL USA; 5 Institute on Aging, University of Florida, Gainesville, FL USA; 6 Department of Radiology, Duke University School of Medicine, Durham, NC USA; 7 Department of Medicine, Duke University School of Medicine, Durham, NC USA

**Keywords:** Lung cancer, Cancer, Oncology

## Abstract

Socioeconomic and racial disparities exist in access to care among patients with non-small cell lung cancer (NSCLC) in the United States. Immunotherapy is a widely established treatment modality for patients with advanced-stage NSCLC (aNSCLC). We examined associations of area-level socioeconomic status with receipt of immunotherapy for aNSCLC patients by race/ethnicity and cancer facility type (academic and non-academic). We used the National Cancer Database (2015–2016), and included patients aged 40–89 years who were diagnosed with stage III-IV NSCLC. Area-level income was defined as the median household income in the patient’s zip code, and area-level education was defined as the proportion of adults aged ≥ 25 years in the patient’s zip code without a high school degree. We calculated adjusted odds ratios (aOR) with 95% confidence intervals (95% CI) using multi-level multivariable logistic regression. Among 100,298 aNSCLC patients, lower area-level education and income were associated with lower odds of immunotherapy treatment (education: aOR 0.71; 95% CI 0.65, 0.76 and income: aOR 0.71; 95% CI 0.66, 0.77). These associations persisted for NH-White patients. However, among NH-Black patients, we only observed an association with lower education (aOR 0.74; 95% CI 0.57, 0.97). Across all cancer facility types, lower education and income were associated with lower immunotherapy receipt among NH-White patients. However, among NH-Black patients, this association only persisted with education for patients treated at non-academic facilities (aOR 0.70; 95% CI 0.49, 0.99). In conclusion, aNSCLC patients residing in areas of lower educational and economic wealth were less likely to receive immunotherapy.

## Introduction

In the United States, lung cancer is the most common cause of cancer death among men and women^1^, with approximately 85% of cases classified as non-small cell lung cancer (NSCLC)^2^. Most patients with NSCLC are diagnosed at an advanced stage, when the cancer is unamenable to curative treatment with surgical resection, and prognosis is poor^2^. Until recently, cytotoxic chemotherapy and targeted therapy were the major types of systemic treatment that prolonged survival among patients with advanced-stage NSCLC (aNSCLC)^3^. In 2015, the Food and Drug Administration (FDA) approved the first use of immunotherapy, a treatment modality that mobilizes the immune system to recognize and destroy cancer cells^3^. Since then, immunotherapy has become a standard modality of treatment for stage IV NSCLC in addition to consolidation therapy after chemoradiation of unresectable stage III disease^3^. This has been shown to be an effective treatment among patients with aNSCLC^4–6^. However, novel therapies, such as immunotherapy, are often cost-prohibitive to those of lower socioeconomic status (SES) and are disproportionately accessible to those with more resources^7^. Indeed, socioeconomic disparities in immunotherapy receipt have been documented for various types of cancers^8–11^. As immunotherapy for NSCLC becomes more widespread in the coming years, characterizing and mitigating disparities in its utilization is paramount.

Beyond understanding overall socioeconomic disparities in immunotherapy utilization, it is also essential to understand the relationship between measures of SES and receipt of immunotherapy among different racial/ethnic groups. In the United States, Black adults are more likely to be of lower SES compared to other racial/ethnic groups^12^. Concurrently, Black patients have lower NSCLC survival as compared to White patients and are often diagnosed at later stages when immunotherapy may be recommended^13–17^. Understanding how socioeconomic factors contribute to treatment receipt among various racial groups is vital for improving aNSCLC survival outcomes, as studies have reported similar survival when patients received equal treatment^18,19^. Healthcare access factors, such as the type of cancer care facility, may influence the treatment types available to and utilized by patients, and thus play a role in outcome disparities^20^. For example, it is well-recognized that patients with cancer treated at academic institutions have better survival outcomes compared to patients treated at community facilities^21,22^.

The purpose of this study was to evaluate the association of area-level SES factors—specifically area-level education and income—with immunotherapy receipt among aNSCLC patients in various racial/ethnic groups. We extend previous research^9^ by evaluating this association in the years immediately after the FDA approval of immunotherapy for NSCLC and stratifying by cancer facility type to understand the role of healthcare access factors. Characterizing this association by race/ethnicity can inform policies that may be useful in mitigating disparities in aNSCLC immunotherapy receipt and consequent NSCLC survival.

## Methods

### Data source

Data for this study were obtained from the 2016 National Cancer Data Base (NCDB) Participant Use File (PUF). The NCDB, a joint project of the American Cancer Society and the Commission on Cancer of the American College of Surgeons, captures 70% of all patients with newly diagnosed cancer in the United States. It contains over 34 million patient records and is the largest clinical registry in the world^23^. Data reported to the NCDB are highly standardized. This study was approved by Duke University Institutional Review Board under a general study protocol (IRB#: Pro00102834) for analyses using NCDB data and was performed in accordance with the Declaration of Helsinki. Because this was a secondary analysis of the NCDB, informed consent from participants was not required.

### Study cohort

**Figure 1:**
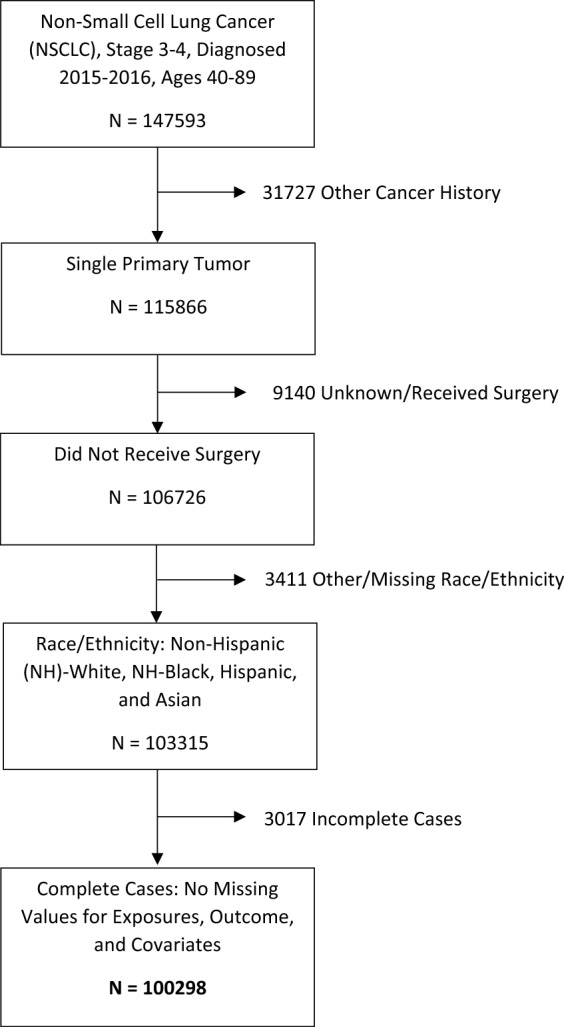
Participant flowchart for advanced-stage non-small cell lung cancer patients in the National Cancer Database.

The present analysis included patients who fit the following criteria: (1) aged 40–89 years; (2) diagnosed with aNSCLC, defined as stage III or IV NSCLC (International Classification of Diseases for Oncology, Third Edition topography codes—C340, C341, C342, C343, C348, and C349) in the years 2015 and 2016; (3) no other cancer history; (4) did not receive surgery; and (5) had no missing values for main study measures or covariates (Fig. 1).

### Study measures and covariates

Immunotherapy receipt was a dichotomized variable: received or not received as first course treatment. First-course treatment in the NCDB refers to treatment that was planned and administered to the patient prior to disease progression or recurrence^23^. Patients’ race/ethnicity was classified into the following groups: Non-Hispanic-White (NH-White), Non-Hispanic-Black (NH-Black), Hispanic, and Asian. Area-level education was specified in the NCDB PUF as the proportion of adults aged 25 or older in the patient’s zip code without a high school degree and categorized as quartiles among all US zip codes: 17.6% or more, 10.9–17.5%, 6.3–10.8%, and < 6.3%. Median household income was similarly estimated by zip code, adjusted for 2016 inflation, and categorized as quartiles: < $40,227, $40,227–$50,353, $50,354–$63,332, > $63,333. The education and income measures were derived by matching the zip code of the patient recorded at the time of diagnosis against files derived from the 2016 American Community Survey data, spanning years 2012–2016. Facility type was categorized into (1) academic and (2) not academic. In addition to these main variables, our analysis also included several covariates: sex, age, comorbidity score (Charlson/Deyo value), year of diagnosis, and primary payer insurance status. Missing values were minimal, amounting to less than 3% for all main study measures and covariates.

### Statistical analysis

The sample was characterized by clinical and demographic characteristics. Assessments of the area-level education and income measures were completed by percent receipt of immunotherapy. The association of area-level education and income with receipt of immunotherapy was evaluated using multilevel hierarchical logistic regression models stratified by race/ethnicity and clustered by facility ID. Receipt of immunotherapy was modeled as the outcome. Each model was adjusted for age, sex, Charlson/Deyo comorbidities score, year of diagnosis, and primary payer insurance status. Model coefficients were transformed to adjusted odds ratios (aOR) and 95% confidence intervals (95% CI). For area-level education models, < 6.3% no high school degree (i.e., high education) was used as the reference group. For area-level income models, median household income > $63,333 (i.e., high income) was used as the reference group. A final set of models were stratified by cancer facility type. Because immunotherapy receipt may be impacted by the presence of comorbidities, we also conducted a sensitivity analysis among patients with a Charlson/Deyo comorbidity score of zero. All analyses were conducted using SAS 9.4 (SAS Institute, Cary, NC, United States).

## Results

**Figure 2:**
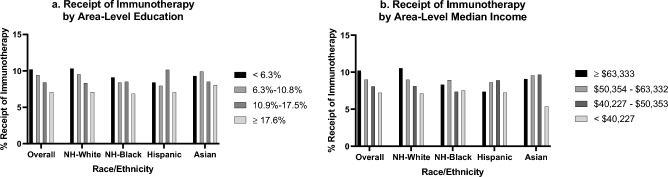
Percent receipt of immunotherapy stratified by race/ethnicity. Area-level education was defined as the proportion of adults aged 25 or older in the patient’s zip code without a high school degree. Area-level median income was defined as the median income in the patient’s zip code.

Of the 100,298 patients with aNSCLC in our analysis, the majority (76%) were 60 years or older at the time of diagnosis (Table 1). Approximately half (54%) of all patients were male and about three-fifths (58%) were Medicare insured. Although about one-fifth (19%) of all patients lived in areas within the highest education quartile, only 7% of NH-Black patients lived in these areas, and only 9% of Hispanic patients. Overall, approximately 9% of all aNSCLC patients received immunotherapy. Those who lived in more educated zip codes were more likely to receive immunotherapy among all patients overall, NH-White patients, and NH-Black patients (Fig. 2). Those who lived in higher income zip codes were more likely to receive immunotherapy among all patients overall and NH-White patients.

**Table 1:** Sociodemographic and cancer characteristics stratified by race/ethnicity for patients with advanced-stage non-small cell lung cancer.

	Overall (%)	Race/ethnicity			
	N = 100,298	NH-White (%) N = 80,212 (80.0)	NH-Black (%) N = 13,150 (13.1)	Hispanic (%) N = 3575 (3.6)	Asian (%) N = 3361 (3.4)
Age					
40–49	3778 (3.8)	2756 (3.4)	553 (4.2)	235 (6.6)	234 (7.0)
50–59	20,513 (20.5)	15,532 (19.4)	3662 (27.9)	688 (19.2)	631 (18.8)
60 +	76,007 (75.8)	61,924 (77.2)	8935 (68.0)	2652 (74.2)	2496 (74.3)
Sex					
Male	54,370 (54.2)	43,214 (53.9)	7227 (55.0)	2099 (58.7)	1830 (54.5)
Female	45,928 (45.8)	36,998 (46.1)	5923 (45.0)	1476 (41.3)	1531 (45.6)
Cancer stage					
III	27,408 (27.3)	22,267 (27.8)	3674 (27.9)	837 (23.4)	630 (18.7)
IV	72,890 (72.7)	57,945 (72.2)	9476 (72.1)	2738 (76.6)	2731 (81.3)
Charlson/Deyo Comorbidity Score					
None	60,623 (60.4)	48,063 (59.9)	7874 (59.9)	2287 (64.0)	2399 (71.4)
1 condition	24,105 (24.0)	19,604 (24.4)	3052 (23.2)	782 (21.9)	667 (19.9)
2 conditions	9616 (9.6)	7853 (9.8)	1284 (9.8)	297 (8.3)	182 (5.4)
≥ 3 conditions	5954 (5.9)	4692 (5.9)	940 (7.2)	209 (5.9)	113 (3.4)
Primary payer insurance					
Not insured	3386 (3.4)	2222 (2.8)	715 (5.4)	310 (8.7)	139 (4.1)
Private insurance/managed care	27,334 (27.3)	21,935 (27.4)	3293 (25.0)	910 (25.5)	1196 (35.6)
Medicaid	9625 (9.6)	6217 (7.8)	2245 (17.1)	648 (18.1)	515 (15.3)
Medicare	58,086 (57.9)	48,313 (60.2)	6618 (50.3)	1666 (46.6)	1489 (44.3)
Other government	1867 (1.9)	1525 (1.9)	279 (2.1)	41 (1.2)	22 (0.7)
Treatment facility					
Academic	31,361 (31.3)	22,724 (28.3)	5688 (43.3)	1463 (40.9)	1486 (44.2)
Not academic	68,937 (68.7)	57,488 (71.7)	7462 (56.8)	2112 (59.1)	1875 (55.8)
Percent of adults in patient zip code without a high school degree quartiles					
< 6.3%	19,465 (19.4)	17,524 (21.9)	856 (6.5)	321 (9.0)	764 (22.7)
6.3–10.8%	28,023 (27.9)	24,342 (30.4)	2206 (16.8)	567 (15.9)	908 (27.0)
10.9–17.5%	29,192 (29.1)	23,402 (29.2)	4331 (32.9)	789 (22.1)	670 (19.9)
≥ 17.6%	23,618 (23.6)	14,944 (18.6)	5757 (43.8)	1898 (53.1)	1019 (30.3)
Median income in patient zip code quartiles					
≥ $63,333	29,298 (29.2)	24,849 (31.0)	1826 (13.9)	828 (23.2)	1795 (53.4)
$50,354–$63,332	23,572 (23.5)	20,071 (25.0)	1903 (14.5)	834 (23.3)	764 (22.7)
$40,227–$50,353	24,666 (24.6)	20,465 (25.5)	2839 (21.6)	876 (24.5)	486 (14.5)
< $40,227	22,762 (22.7)	14,827 (18.5)	6582 (50.1)	1037 (29.0)	316 (9.4)
Immunotherapy receipt					
Received	8748 (8.7)	7136 (8.9)	1026 (7.8)	286 (8.0)	300 (8.9)
Not received	91,550 (91.3)	73,076 (91.1)	12,124 (92.2)	3289 (92.0)	3061 (91.1)

**Figure 3:**
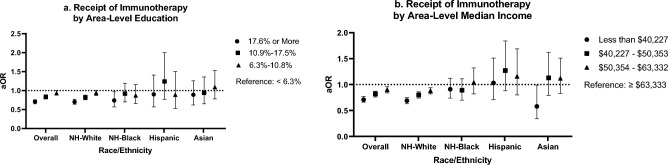
Multivariable analyses evaluating associations of area-level socioeconomic factors with receipt of immunotherapy, stratified by race/ethnicity. Area-level education was defined as the proportion of adults aged 25 or older in the patient’s zip code that did not graduate from high school. Area-level median income was defined as the median income in the patient’s zip code. Estimates were adjusted for age, insurance status, Charlson/Deyo comorbidities score, year of diagnosis, and sex. *aOR* adjusted odds ratio.

In multivariable analyses, (Fig. 3; Table 2), we found that those living in neighborhoods with the lowest education levels were 29% less likely to receive immunotherapy relative to their counterparts living in the most educated areas (aOR 0.71; 95% CI 0.65, 0.76). When stratified by race/ethnicity, this association was similar for NH-White aNSCLC patients (aOR 0.70; 95% CI 0.64, 0.77), and among NH-Black patients (aOR 0.74; 95% CI 0.57, 0.97). Furthermore, patients within the lowest income areas were 29% less likely to receive immunotherapy (aOR 0.71; 95% CI 0.66, 0.77). In race-stratified analyses, we continued to observe a similar association among NH-White aNSCLC patients (aOR 0.69, 95% CI 0.63, 0.75). However, income level was not significantly associated with immunotherapy receipt among NH-Black patients (aOR 0.91; 95% CI 0.74, 1.12). Our sensitivity analysis among patients with a Charlson/Deyo comorbidity score of zero yielded similar results (data not shown).

**Table 2:** Multivariable analyses evaluating associations of area-level socioeconomic factors with receipt of immunotherapy, stratified by race/ethnicity.

	Overall	Race/ethnicity			
		NH-White	NH-Black	Hispanic	Asian
Percent of adults in patient zip code without a high school degree quartiles					
< 6.3% (Ref.)	#	#	#	#	#
6.3–10.8%	**0.93 (0.88, 1.00)**	0.94 (0.87, 1.00)	0.87 (0.66, 1.16)	0.89 (0.53, 1.50)	1.09 (0.78, 1.53)
10.9–17.5%	**0.83 (0.78, 0.89)**	**0.82 (0.76, 0.88)**	0.92 (0.70, 1.19)	1.24 (0.77, 2.00)	0.94 (0.65, 1.36)
≥ 17.6%	**0.71 (0.65, 0.76)**	**0.70 (0.64, 0.77)**	**0.74 (0.57, 0.97)**	0.90 (0.57, 1.41)	0.89 (0.62, 1.26)
Median income in patient zip code quartiles					
≥ $63,333 (Ref.)	#	#	#	#	#
$50,354–$63,332	**0.90 (0.84, 0.96)**	**0.88 (0.82, 0.94)**	1.04 (0.82, 1.32)	1.16 (0.80, 1.69)	1.12 (0.83, 1.51)
$40,227–$50,353	**0.82 (0.76, 0.87)**	**0.80 (0.74, 0.86)**	0.89 (0.70, 1.11)	1.27 (0.88, 1.84)	1.13 (0.79, 1.62)
< $40,227	**0.71 (0.66, 0.77)**	**0.69 (0.63, 0.75)**	0.91 (0.74, 1.12)	1.03 (0.71, 1.51)	**0.58 (0.34, 0.99)**

Area-level education was defined as the proportion of adults aged 25 or older in the patient’s zip code without a high school degree. Area-level median income was defined as the median income in the patient’s zip code.

Estimates were adjusted for age, Charlson/Deyo comorbidities score, insurance status, year of diagnosis, and sex.

*aOR* adjusted odds ratio.

Bold indicates p < 0.05.

**Figure 4:**
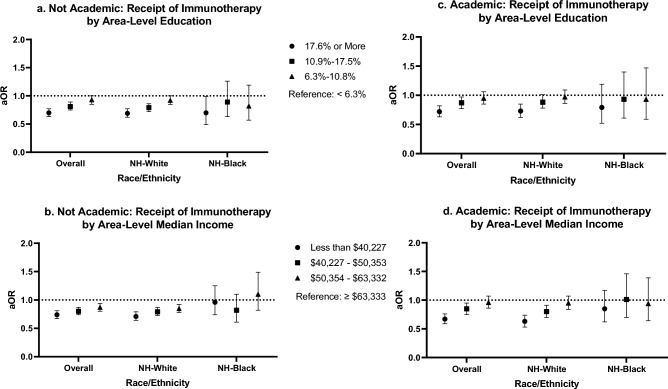
Multivariable analyses evaluating associations of area-level socioeconomic factors with immunotherapy receipt, stratified by race/ethnicity and cancer facility type. Area-level education was defined as the proportion of adults aged 25 or older in the patient’s zip code without a high school degree. Area-level median income was defined as the median income in the patient’s zip code. Estimates were adjusted for age, insurance status, Charlson/Deyo comorbidities score, year of diagnosis, and sex. *aOR* adjusted odds ratio.

Among those treated at non-academic facilities, living in the least educated areas compared to the most educated areas was associated with 30% decreased odds of receiving immunotherapy (aOR 0.70; 95% CI 0.63, 0.77) (Fig. 4; Table 3). Among those treated at academic cancer facilities, living in the least vs. most educated areas was associated with 28% decreased odds (aOR 0.72; 95% CI 0.63, 0.82). A similar association was observed among NH-White patients treated at non-academic facilities (aOR 0.69; 95% CI 0.62, 0.77) and NH-White patients treated at academic facilities (aOR 0.73; 95% CI 0.62, 0.85). Among NH-Black patients who were treated at academic facilities, residing in the least educated areas was associated with 30% decreased odds of receiving immunotherapy (aOR 0.70; 95% CI 0.49, 0.99). A similar pattern was observed for living in regions with the lowest area-level income vs. highest area-level income for all patients overall (non-academic: aOR 0.74; 95% CI 0.67, 0.81 and academic: aOR 0.67; 95% CI 0.59, 0.76) and NH-White patients (non-academic: aOR 0.71; 95% CI 0.64, 0.79 and academic: 0.63; 95% CI 0.53, 0.74). However, no associations were observed for NH-Black patients regardless of cancer facility type (non-academic: aOR 0.96; 95% CI 0.74, 1.25 and academic: aOR 0.85; 95% CI 0.62, 1.17).

**Table 3:** Multivariable analyses evaluating associations of area-level socioeconomic factors with immunotherapy receipt, stratified by race/ethnicity and cancer facility type.

	Overall	Race/ethnicity	
		NH-White	NH-Black
Percent of adults in patient zip code without a high school degree quartiles 2012–2016: ≥ 17.6% vs. < 6.3% (Ref.)			
Academic	**0.72 (0.63, 0.82)**	**0.73 (0.62, 0.85)**	0.79 (0.52, 1.19)
Not Academic	**0.70 (0.63, 0.77)**	**0.69 (0.62, 0.77)**	**0.70 (0.49, 0.99)**
Median income in patient zip code quartiles 2012–2016: < 40,227 vs. ≥ $63,333 (Ref.)			
Academic	**0.67 (0.59, 0.76)**	**0.63 (0.53, 0.74)**	0.85 (0.62, 1.17)
Not Academic	**0.74 (0.67, 0.81)**	**0.71 (0.64, 0.79)**	0.96 (0.74, 1.25)

Area-level education was defined as the proportion of adults aged 25 or older in the patient’s zip code without a high school degree. Area-level median income was defined as the median income in the patient’s zip code.

Estimates were adjusted for age, Charlson/Deyo comorbidities score, insurance status, year of diagnosis, and sex.

*aOR* adjusted odds ratio.

Bold indicates p < 0.05.

## Discussion

In this analysis of aNSCLC patients in the NCDB, we observed that patients living in regions of lower area-level income and education were less likely to receive immunotherapy treatment in the years immediately following its approval. Among NH-Black patients, this association persisted only for area-level education. When we stratified by facility type, lower area-level income and education were associated with lower likelihood of immunotherapy treatment regardless of facility type among NH-White patients. However, among NH-Black patients, a significant association was only observed with education for patients treated at non-academic facilities. Our results indicate underlying disparities in access to high-quality cancer treatment by SES in the United States.

Consistent with our findings, several studies have shown that patients living in socioeconomically deprived areas are less likely to receive immunotherapy for many different cancer types^8–11^. In a study of stage IV NSCLC patients diagnosed from 2004–2015, receipt of immunotherapy-like compounds was more common among healthier patients (lower Charlson/Deyo value) and those living in more educated regions and less common among Black patients^9^. Additionally, research among metastatic melanoma patients found that Black patients living in less educated and lower income areas were less likely to receive immunotherapy, although immunotherapy was associated with improved overall survival^8,10^. In a study among patients with hepatobiliary cancer, most patients who received immunotherapy lived in higher income areas and were treated at academic facilities^11^. Furthermore, for NSCLC, several studies have demonstrated associations between measures of SES and timeliness of care and treatment utilization^24–26^.

In the context of immunotherapy and precision medicine more broadly, a systematic review found that there are significant socioeconomic inequalities in biological and precision therapy utilization, reporting that lower SES patients were 17% less likely to be treated with precision medicine therapies^27^. Genetic testing is often required before immunotherapy can be provided^28^, and socioeconomic disparities in receipt of genetic testing have been documented as well^29^. Importantly, studies have argued that novel cancer therapies, such as immunotherapy, disproportionately favor those with more resources, potentially contributing to and widening inequalities in cancer care and treatment^30,31^. Efforts to address socioeconomic inequities along each step of the pathway to receiving immunotherapy and other forms of precision medicine are urgently needed.

Our results by cancer facility type show that disparities in receipt of immunotherapy by income and education exist among NH-White patients treated at both academic and non-academic facilities. Similar results were observed among NH-Black patients for area-level education; however, statistical significance was only achieved for those treated at non-academic facilities. Academic facilities are typically more well-resourced than community facilities, and patients are more likely to have access to specialists and should experience better outcomes regardless of socioeconomic resources^32–35^. However, our results and prior studies suggest that concerted efforts are needed to ensure equity even in these high-resourced facilities^36,37^. Beyond influencing the affordability of care, higher SES, and education in particular, may lead to positive feelings of belonging and familiarity within academic facilities and healthcare settings at-large, and lower levels of distrust^38,39^. High-quality patient-provider communication is critical to strengthening relationships with local communities and ensuring that all patients can benefit from advances in cancer care^40^. Further research is warranted to delineate pathways for improving equity among patients with low SES in the United States, specifically in the early years following the approval of novel therapeutics.

Our study has several limitations that should be considered when contextualizing our results. First, only first-course treatments, which refer to those treatments that were planned and administered to the patient prior to disease progression or recurrence, are recorded in the NCDB^23^. Therefore, if immunotherapy was given later, it may not be recorded in the database. Second, similar to many analyses using large databases, we used area-level measures of education and income. These measures may not precisely represent each patient’s individual circumstances; however, they are important in capturing the context in which patients make decisions because where patients live often shapes the care available to them. Third, the NCDB also does not note specific types of immunotherapy regimens or additional details, such as the duration of therapy or molecular biomarkers, including PDL1 and driver mutation status (i.e., EGFR, ALK, etc.). Finally, because the NCDB contains consolidated data from various reporting sites, there may be some inconsistency in the definition of variables such as race/ethnicity. Differences in data collection methods of race/ethnicity or interpretation of race/ethnicity among providers may introduce misclassification bias. However, our study also has important strengths including a large sample size and stratification by race and cancer facility type.

In conclusion, we show that lower area-level income and education is associated with a lower likelihood of receiving immunotherapy among aNSCLC patients. There is an urgent need to develop strategies to provide equitable access to immunotherapy. Detailed insights into social inequities in the context of cancer care will inform the development of interventions to ensure appropriate receipt of novel cancer treatments, such as immunotherapy, with the goal of improving survival and outcomes of patients with aNSCLC.

## Data Availability

The data used in this study are available from the National Cancer Database (https://www.facs.org/quality-programs/cancer/ncdb).
